# Dr. Bennet Dowler

**Published:** 1859-08

**Authors:** 


*Dr. Bennet Dowler.*—In looking over the French periodical medical literature, we are gratified to see that American *savans* are now playing a more important part than formerly in foreign appreciation of scientific matters. One of the best known in Paris of our eminent men, is Dr. Bennet Dowler, the distinguished experimental physiologist and pathologist of New Orleans. In the No. of the *Gazette Hebdomadaire* for May 27, we have an elaborate report on Dr. Dowler's memoir entitled, "A contribution to the pathological anatomy of the large intestine and the fœcal retention," to the "*Société de Médicine de la Seine*," by Dr. Auguste Voisin. This report speaks in a most complimentary manner of the value of the labors of Dr. Dowler.

Experimental physiology, now exciting so much attention on both sides of the Atlantic, was investigated early in this country by Dr. Dowler, who produced important results in the physiology of the nervous system, and elucidated many points in the mechanism of the capillary circulation before involved in obscurity. Dr. Dowler has also distinguished himself in the onerous and responsible duties of editor of the *New Orleans Medical and Surgical Journal*, a situation which called forth his accomplishments in the general literature of the profession, and added to the fame which he had already acquired as a thorough and investigating physiologist. A just and gratifying tribute is paid to Dr. Dowler, under its appropriate head, in the "*New American Cyclopædia.*" The career of such a man in the beautiful study of physiology, especially in its recent mode of investigation, is an encouraging example to younger students, to whom this field is now especially open; few, however, can hope to attain the reputation which his talents and industry have commanded.

